# Arrhythmia Diagnosis by Using Level-Crossing ECG Sampling and Sub-Bands Features Extraction for Mobile Healthcare

**DOI:** 10.3390/s20082252

**Published:** 2020-04-16

**Authors:** Saeed Mian Qaisar, Syed Fawad Hussain

**Affiliations:** 1 College of Engineering, Effat University, Jeddah 22332, Saudi Arabia; 2 Machine Learning and Data Science Lab, Ghulam Ishaq Khan Institute of Engineering Sciences and Technology, Topi 23460, Pakistan; fawadhussain@giki.edu.pk

**Keywords:** electrocardiogram (ECG), compression, arrhythmia classification, level-crossing sampling, adaptive-rate processing, hysteresis, machine learning, mobile healthcare, wavelet decomposition, sub-bands features extraction

## Abstract

Mobile healthcare is an emerging technique for clinical applications. It is usually based on cloud-connected biomedical implants. In this context, a novel solution is presented for the detection of arrhythmia by using electrocardiogram (ECG) signals. The aim is to achieve an effective solution by using real-time compression, efficient signal processing, and data transmission. The system utilizes level-crossing-based ECG signal sampling, adaptive-rate denoising, and wavelet-based sub-band decomposition. Statistical features are extracted from the sub-bands and used for automated arrhythmia classification. The performance of the system was studied by using five classes of arrhythmia, obtained from the MIT-BIH dataset. Experimental results showed a three-fold decrease in the number of collected samples compared to conventional counterparts. This resulted in a significant reduction of the computational cost of the post denoising, features extraction, and classification. Moreover, a seven-fold reduction was achieved in the amount of data that needed to be transmitted to the cloud. This resulted in a notable reduction in the transmitter power consumption, bandwidth usage, and cloud application processing load. Finally, the performance of the system was also assessed in terms of the arrhythmia classification, achieving an accuracy of 97%.

## 1. Introduction

An electrocardiogram (ECG) signal possesses critical information about cardiac functionality [1]. An abnormality of cardiac rhythm is a sign of certain diseases that can be diagnosed by an effective analysis of an ECG [2]. Heart diseases are one of the major threats to human life [3], and a timely diagnosis can lead to better measures. To do this, individual heartbeats are analyzed by exploiting their frequency content and morphological patterns for the automatic diagnosis of ECG arrhythmia [4,5,6,7,8]. Interferences and physiological artifacts can modify the ECG signal, thus decreasing the effectiveness of the automatic diagnosis mechanism. To overcome these deficiencies, numerous signal processing techniques have been used including eigenvalue composition [9], extended Kalman filtering [10], and Fourier decomposition [11]. The denoised ECG signals are processed and analyzed to extract classifiable features that can assist in automatic diagnosis. Some broadly used features extraction techniques are wavelet packet decomposition (WPD) [4], wavelet-based kernel principle component analysis (wkPCA) [4], wavelet packet entropy (WPE) [5], discrete wavelet transform (DWT) [6,12,13], temporal and morphological features [12], principle component analysis (PCA) [13,14], bispectrum [14], and Hermite function coefficient and temporal features [15].

The extracted features are employed for the automatic diagnosis of cardiac diseases. Supervised machine learning techniques are quite popular in these applications. In this regard, numerous approaches have been presented for ECG signal classification including those of the back propagation neural network (BNN) [4], random forest (RF) [5], probabilistic neural network (PNN) [6], support vector machine (SVM) [12], support vector machine (SVM)-radial basis function (RBF) [13,14], and optimized block-based neural network (OBNN) [15].

A timely detection of arrhythmia conditions allows for effective cardiac failure treatment. Therefore, patients with cardiac problems require continuous monitoring. In this context, wearable ECG sensors have been used [16,17]. These are usually linked to the cloud, and their data are logged and further processed via cloud-based applications. The decision support outcome is then shared with a monitoring center [17,18,19,20]. Such an approach allows healthcare specialists to make timely decisions and offer emergency care for patients with chronic disorders.

The design of ECG wearable devices is challenging because of strict constraints on size, weight, and power consumption. Self-powered wireless sensors are preferred because they enable the acquisition of ECG signals without causing too much uneasiness to the patient. A precise diagnosis requires the fine-grained recording and analysis of multichannel ECG signals. Consequently, the data dimension is exponentially augmented [21]. The transmission, analysis, and storage of such a big amount of data are not efficient. The substantial saving of power in a wireless ECG device is only possible by minimizing the activity of acquisition, processing, and wireless data transmission. In this context, studies have been carried out on ECG signal compression [20,21,22], non-uniform sampling [16,23,24], and adaptive-rate processing [25].

Conventional ECG processing solutions are based on the Nyquist sampling theory. Systems are time-invariant, which results in a worst-case parameterization [25,26,27,28,29]. A system’s computational load and processing activity remain fixed regardless of the incoming ECG signal time-varying nature. As a result, the system is constrained. It captures and processes redundant samples that increase the system’s overall computational load, power consumption, processing, and transmission activities [27,29]. These shortfalls can be compensated for to a certain extent by using level-crossing analog-to-digital converters (LCADCs) [16,24,30,31,32,33,34,35]. These converters adapt the system acquisition and processing rates according to the incoming signal’s temporal variations, a process that renders a significant computational efficiency of the suggested approach compared to traditional ones. In [27,29], Qaisar et al. reported more than one order of magnitude gain in terms of computational outperformance compared to the classical counterparts.

This work contributes to the development of efficient automatic arrhythmia diagnosis in the mobile healthcare framework. The aim is to realize an effective solution by achieving real-time compression and efficient ECG signal processing and transmission. In a continuation of previous works, such as [16,24,28,30,31], level-crossing sampling and adaptive-rate processing techniques are proposed in this paper. The realization of such is achieved by using an efficient assembly of the LCADC, robust classifiers, adaptive-rate signal processing, and features extraction modules. The use of level-crossing sampling and adaptive-rate processing for arrhythmia detection is an original concept thatis explored in this paper.

## 2. Materials and Methods

**Figure 1:**
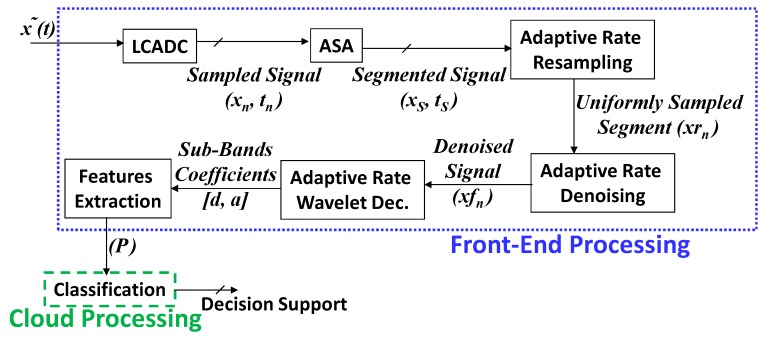
The proposed system block diagram. LCADC: level-crossing analog-to-digital converters; ASA: activity selection algorithm.

The proposed framework is shown in Figure 1, where the dotted blue-colored border (‘….’) encloses blocks embedded in the ECG real-time wearable processing device. The cloud processing module is enclosed in a green-colored dashed line (‘---’).

### 2.1. Dataset

The performance of the designed solution was studied by using the MIT-BIH arrhythmia database (ECG arrhythmia time series are available under (https://physionet.org/content/mitdb/1.0.0/)) [36]. A set of twelve 30-min ECG recordings containing clinically important arrhythmias was employed. Each channel was band-limited between [0.5, 60] Hz by using an analog antialiasing filter and was then acquired with an 11-bit resolution analog-to-digital converter (ADC). The sampling rate was 360 Hz. Experienced cardiologists labeled all acquired heartbeats. To extract individual heartbeats, each considered ECG signal wassegmented for a time length of 0.9 s. Five different arrhythmias classes were considered: normal signals (N), right bundle branch block (RBBB), left bundle branch block (LBBB), atrial premature contraction (APC) and premature ventricular contraction (PVC). To achieve equal representation, 300 instances were considered for each class. Therefore, in total, there were 1500 instances, each belonging to one of the five considered classes. To avoid any bias, heartbeats related to each class were collected from various records (clear from Table 1).

### 2.2. Level-Crossing A/D Converter (LCADC)

To evaluate the LCADC, the sampled version of a band-limited signal, given by $y\left(t_{n}\right)$ and obtained from the MIT-BIH dataset, was reconstructed. If $\tilde{x}\left(t\right)$ is the reconstructed quasi-analog version of $y\left(t_{n}\right)$, then the relationship between $\tilde{x}\left(t\right)$ and $y\left(t_{n}\right)$ can be mathematically presented by using Equation (1), where U is the up-sampling factor. The up-sampling is realized by using the standard cascaded cubic spline interpolators and anti-imaging filters [37].

(1) $$\tilde{x}\left(t\right)=y\left(t_{\frac{n}{U}}\right).$$

**Figure 2:**
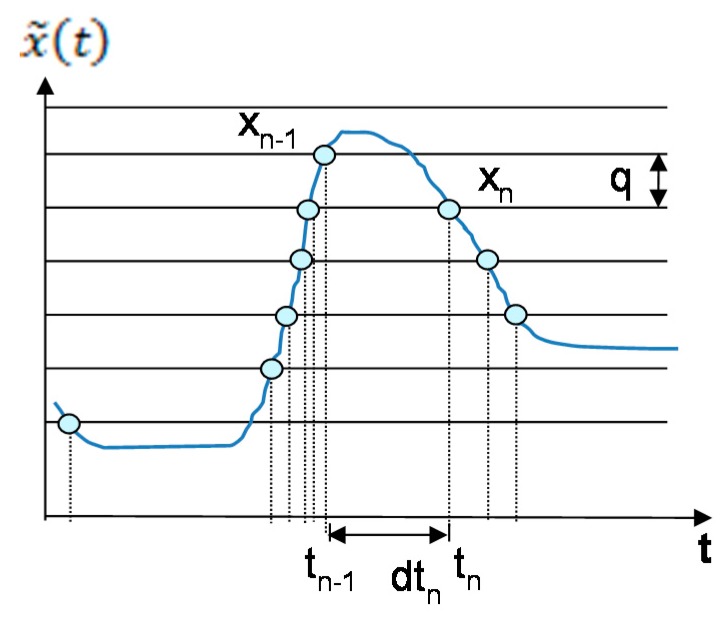
The principle of a level-crossing ADC with hysteresis.

The band-limited signal $\tilde{x}\left(t\right)$ is digitized with a LCADC. The frequency content of $\tilde{x}\left(t\right)$ is limited to [0.5, 60] Hz [9,36]. The LCADC is designed on the basis of level-crossing sampling (LCS) [28,38]. In this case, a sample is only acquired when $\tilde{x}\left(t\right)$ crosses one of the predefined thresholds. For a given LCADC amplitude dynamic, ΔV, and resolution, M, the sampling frequency is piloted by the signal. Samples are irregularly spaced in time, and the count of samples is proportional to the slope of $\tilde{x}\left(t\right)$ [31]. Sample amplitudes are equal to the predefined thresholds. The sampling instants are defined by Equation (2), and the process is shown in Figure 2 [28,38], where $t_{n}$ is the present sampling instant, $t_{n-1}$, is the preceding one and the time step between the present and the preceding sampling instants is $dt_{n}$. LCADC parameters are selected according to the approach described in [35]. This approach is based on the uniform-quantization scheme. Therefore, its quantum, q, can be calculated as: $q=\frac{\Delta V}{2^{M-1}}$ [38].

(2) $$t_{n}=t_{n-1}+dt_{n}.$$

The phenomenon of hysteresis is also embedded in the LCADC [29]. A new sample is only acquired when there is a difference of q with respect to the preceding sample amplitude. The process is shown in Figure 2 and can be mathematically expressed as: $x_{n}=x_{n-1}\pm q$. It improves LCADC efficiency in terms of real-time compression [29]. The QRS complexes of heartbeats contain the most significant arrhythmia-related information [16,24]. The LCADC acquires the relevant ECG information, QRS complexes, at adaptive-rates while avoiding the remaining low amplitude components [24]. Therefore, it collects a reduced number of samples while comparing them with the classical counterparts [24].

The working principle of an LCADC is different from conventional ADCs [38]. While considering the ideal case, for conventional ADCs, the sampling instants are accurately known. However, the amplitudes of samples are quantized [38]. Quantization is the only source of error, and it depends on the selected ΔV and M [35]. It is assessed in terms of the signal-to-noise ratio (SNR) [38]. The SNR is computable as: $SNR_{dB}=6.02M+1.76$. It presents an ideal ADC SNR for a full-scale monotonous sinusoidal input and describes its dependency on M. On the contrary, the amplitudes of samples are ideally known for an LCADC. However, the instants of these samples are quantized according to the operating frequency, $F_{Timer}$, of the timer circuit thatis used to record these instants [28]. The SNR of an ideal LCADC can be calculated by using Equation (3) [38], where $f_{sig}$ is the frequency of the full-scale sinusoid used to evaluate the LCADC. Equation (3) shows that the SNR of an ideal LCADC is independent of M and is a function of $f_{sig}$ and $T_{Timer}=\frac{1}{F_{Timer}}$. A 6.02 dB improvement in the value of the SNR is achievable by halving *T_timer_* [28]. In this study, a 21-bit resolution timer wasused with $F_{Timer}$ = 1 MHz. These parameters allowed us to properly record a heartbeat without timer overflow and resulted in an ideal LCADC SNR of 73.25 dB. According to [4,5,6,12,13,14,15], an11-bit ADC resolution is appropriate and results in a precise, computer-based arrhythmia diagnosis. For the selected timer parameters, the obtained LCADC SNR wasequal to the theoretical SNR of an 11.9-bit classical ADC. This justified the selected system parameters for the considered application.

(3) $$SNR_{dB}=-11.19-20log\left(f_{sig}\cdot T_{timer}\right)$$

### 2.3. Activity Selection Algorithm (ASA)

**Figure 3:**
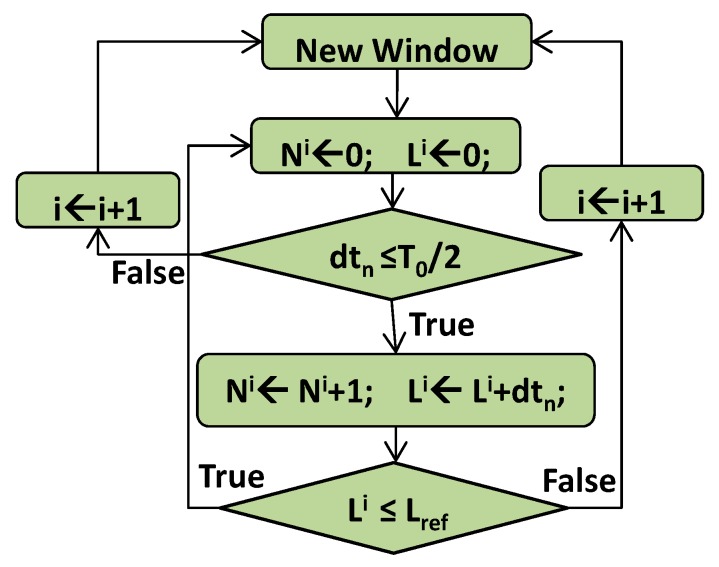
The algorithmic state machine (ASM) chart of activity selection algorithm.

The activity selection algorithm (ASA) segments LCADC output [27,28]. Thisalgorithmemploys sampling process non-uniformity for selecting the active parts of the signal while avoiding the redundant baseline [16,24,28]. The principle is clear from the algorithmic state machine (ASM) chart shown in Figure 3. In Figure 3, $T_{0}=\frac{1}{f_{min}}$ is the fundamental period of $\tilde{x}\left(t\right)$, and $f_{min}$ is the lowest frequency component whose value is equal to 0.5 Hz [9,36]. The level-crossing concept based sampled signal active parts are identified by using values of $T_{0}$ and $dt_{n}$. The condition $dt_{n}\leq T_{0}$ is selected to respect the Nyquist criterion for $f_{min}$. $L^{i}$ is the length of the *i^th^* selected segment $W^{i}$. $N^{i}$ is the number of samples for $W^{i}$. $L_{ref}$ is the superior bound on $L^{i}$, and its choice depends on the system parameters and the characteristics of the intended signal [28,38]. For this study $L_{ref}$ = 1-swas selected. At the beginning of each iteration, ‘*i*’ is incremented, and $N^{i}$ and $L^{i}$ are initialized to zero.

The traditional windowing functions do not provide interesting features of the ASA [27,29]. The ASA allows for the selection of the signal active portions while avoiding the redundant, unwanted ones [16,24,28]. In addition, the length of the window-function is automatically adjusted according to the temporal variations of the signal. This process avoids signal truncation, and, therefore, segmentation can be performed by using adaptive length rectangular windows. This avoids the use of arithmetically complex smoothening-window functions and creates an effective solution of the spectral leakage phenomenon [27].

### 2.4. Adaptive-Rate Resampling

For a given resolution *M*, the LCADC sampling frequency organizes by following the temporal variations of $\tilde{x}\left(t\right)$. The maximum sampling frequency, $Fs_{max}$, of a uniform quantization-based LCADC is defined by Equation (4) [28,38], where *f_max_* is the $\tilde{x}\left(t\right)$ bandwidth. *A_in_* is the input signal amplitude.

(4) $$Fs_{max}=2\cdot f_{max}\left(2^{M}-2\right)\cdot \frac{A_{in}}{\Delta V}$$

The sampling frequency for $W^{i}$ can be calculated as: $Fs^{i}=\frac{N^{i}}{L^{i}}$. To benefit from the established signal processing techniques, $W^{i}$ is uniformly resampled by using simplified linear interpolation (SLI) [29], which modifies the resampled signal compared to the original; this variation depends on M, q, and the employed interpolator [39]. For SLI, the superior bound of error per resampled observation is $\frac{q}{2}$ [39].

The LCADC focuses on the active signal parts. Nevertheless, one LCADC shortfall is that the active signal parts can be digitized at superior rates compared to conventional digitization approaches [27,29]. The ASA overcomes this shortfall by examining features of $W^{i}$ and thenadjusting the system parameters accordingly [29]. In this way, the resampling frequency, $Frs^{i}$, and the arithmetic complexity of the post-processing modules are adjusted by following the $\tilde{x}\left(t\right)$ temporal disparities [29].

### 2.5. Adaptive-Rate Denoising

A band-pass finite impulse response (FIR) filters bank is designed for the effective online diminishing of unwanted noise from ECG signals [9]. The FIR filtering process can be mathematically described by using Equation (5), where $x_{n}$ is the incoming signal, $xf_{n}$ is the filtered signal, and $h_{k}$ are the coefficients of the $K^{th}$ order FIR filter.

(5) $$xf_{n}=\overset{K-1}{\underset{k=0}{\sum }}h_{k}\times x_{n-k}.$$

Here, the filters bank was designed for the cut-off frequencies of [${\mathrm{Fc}}_{\min}$, ${\mathrm{Fc}}_{\max}$] Hz. Each filter was implemented for a different sampling frequency that was chosen from the set Fref (cf. Equation (6)). The upper bound on *Fref* was selected as *F_r_* and, to assure a proper digital filtering operation, the lower bound on *Fref* was chosen as *Fs_min_* ≥ 2·*F_Cmax_* [29]. *F_r_* was the sampling frequency such that its value remained greater than and closer to *F_Nyq_* = 2·*F_Cmax_*. *Q* is the length of *Fref*, and its value is always chosen as a binary-weighted. In Equation (6), Δ is a unique offset and can be computed as: Δ=$\frac{F_{r}-Fs_{min}}{Q-1}$.

(6) $$Fref=\left\{Fs_{\min},Fs_{\min}+\Delta ,\ldots ,Fs_{\min}+\left(Q-1\right)\Delta =F_{r}\right\}$$

**Figure 4:**
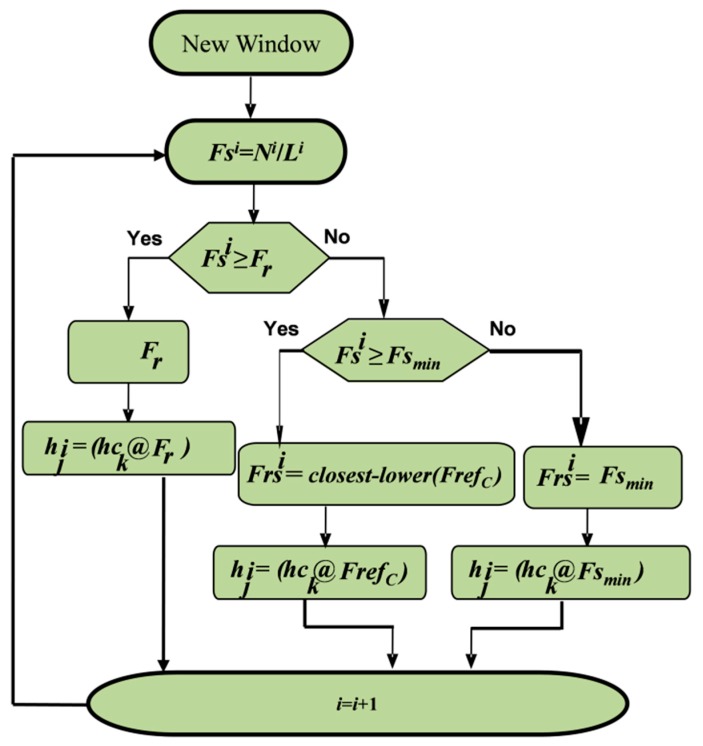
The process of selecting the resampling frequency and the filter coefficients from the reference filter bank for the *i^th^* selected segment.

The ASA examines the properties of $W^{i}$ and uses them for adjusting the denoising parameters such as the resampling frequency, $Frs^{i}$, and the filter order, $K^{i}$. The method of choosing $Frs^{i}$ and keeping it aligned with $Fref_{C}$ is shown in Figure 4, which shows that a suitable filter, from the reference bank, is selected for each $W^{i}$. Let $h_{c_{k}}$ be the selected filter for $W^{i}$ that is sampled at $Fref_{C}$. Then, this selection can be made on the basis of Fref and $Fs^{i}$. For proper denoising, $Frs^{i}$ = $Fref_{C}$ must bechosen [29].

### 2.6. Features Extraction

#### 2.6.1. Adaptive-Rate Discrete Wavelet Transform (ARDWT)

The wavelet transform (WT) is frequently used for the multi-resolution time-frequency analysis of non-stationary ECG like signals [4,40]. This transform can be mathematically expressed by Equation (7), where *s* and *u*, respectively, represent the dilation and the translation parameters.

(7) $$W_{x}^{\psi }\left(u,s\right)=\frac{1}{\sqrt{S}}\int_{-\infty }^{+\infty }x\left(t\right)\psi *\left(\frac{\left(t-u\right)}{s}\right)dt$$

**Figure 5:**
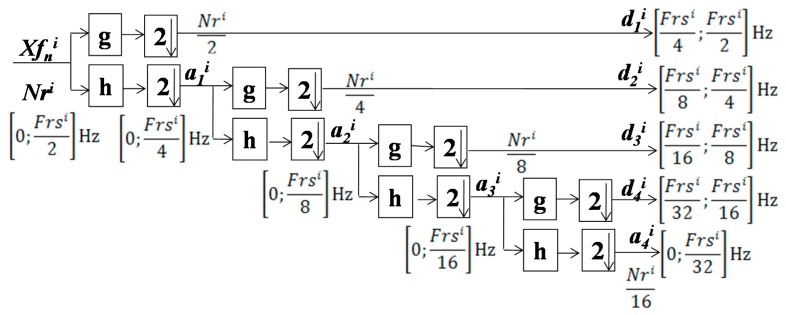
The adaptive-rate discrete wavelet transform (ARDWT) scheme with four levels of decomposition.

A discrete-time wavelet transform (DWT) is used for analyzing digital signals. A translation-dilation representation is attained by employing digital filters. In this case, a denoised segment, $W^{i}$, is passed through the Daubechies algorithm-based wavelet decomposition, which consists of half-band high-pass and low-pass filters. This allows for the computation of approximation, $a_{m}^{i}$, and detail, $d_{m}^{i}$, coefficients at each level of decomposition. The mathematical processes of computing $a_{m}^{i}$ and $d_{m}^{i}$ are, respectively, expressed by Equations (8) and (9), where m represents the level of decomposition. In this study, a fourth level of decomposition was performed, i.e., m∈{1,2,3,4}. $g_{2n-k}$ and $h_{2n-k}$ are, respectively, the low-pass and high-pass FIR filters with a subsampling factor of 2. The process is further illustrated in Figure 5.

According to [40], sub-bands extracted by wavelet decomposition are functions of the incoming signal sampling frequency. In the proposed solution, the $W^{i}$ can be resampled at a specific frequency $Frs^{i}$, resulting in an adaptive-rate decomposition for each $W^{i}$, potentially achievingsub-bands with a lesser computational cost. This happens because the system has to process fewer number of samples compared to fix-rate decomposition concepts [4,5,6,12,13,14,15]. Furthermore, the adjustment of $Frs^{i}$ allows for a better focus on the incoming signal band of interest compared to the fix-rate counterparts [4,5,6,12,13,14,15].

(8) $$a_{m}^{i}=\overset{K_{g}^{i}}{\underset{k=1}{\sum }}xf_{n}^{i}\cdot g_{2n-k}$$

(9) $$d_{m}^{i}=\overset{K_{g}^{i}}{\underset{k=1}{\sum }}xf_{n}^{i}\cdot h_{2n-k}$$

#### 2.6.2. Features

The wavelet coefficients obtained for each intended sub-band—$d_{1}^{i}$, $d_{2}^{i}$, $d_{3}^{i}$, $d_{4}^{i}$, and $a_{4}^{i}$—were used for extracting classifiable signal features. Nine statistical features were extracted for each considered sub-band. Therefore, in total, 45 features were employed to represent each selected segment.

The same features were extracted from each considered sub-band, as listed below.

*Power Spectrum of the Signal (PS)*

Power spectrum (PS) is computed as the average absolute value of the spectral means.

*Mean Absolute Value of the Signal (MAV)*

The mean absolute value (MAV) is calculated by adding all the absolute values of coefficients and then normalizing the sum.

*Standard Deviation (STD)*

Standard deviation (STD) is the measure of intended coefficients dispersion from the mean value.

*Skewness of the Signal (SK)*

Skewness (SK) is a measure of the asymmetry of the frequency distribution around its mean.

*Kurtosis of the Signal (K)*

Kurtosis (K) is a measure of the curvature of the considered coefficients.

*Mean Ratio (R)*

The mean ratio (R) is the ratio of the mean value of the detailed signal to the mean value of the approximate signal.

*Peak Positive Value (PV)*

The peak positive value (PV) is the maximum positive amplitude of the considered coefficients.

*Peak Negative Value (NV)*

The peak negative value (NV) is the maximum negative amplitude of the considered coefficients.

*Second Peak Negative Value (NV2)*

The second peak negative value (NV2) is the second maximum negative amplitude of the considered coefficients.

### 2.7. Classification Methods

Here, once relevant features were extracted, the data were represented in the form of a reduced data matrix, composed of 45 features, with 9 from each of the 5 selected sub-bands for each intended instance. Since the employed dataset had 5 ECG classes with300 instances per class, the resulting data matrix had a size of 1500 × 45. To classify this data matrix, the following classification techniques were employed.

Several of the used classification techniques require parameters tuning. To do this, we used the standard method of validation to tune parameters during the training phase to get the appropriate average result. These values were then fixed for the testing phase and are mentioned below.

#### 2.7.1. *k*-Nearest Neighbors (*k*-NN)

The *k*-nearest neighbors (*k*-NN) algorithm [41,42] uses the *k* nearest neighbors of a test sample from the training dataset. Let *v_j_* represent a sample and <*v_j_*, *l_j_*> denote a tuple of a training sample and its label, *l_j_* ∈ [1,*C*] where *C* is the number of classes. Given a test sample, *z*, the mathematical process of computing the nearest neighbor, *j*, is presented by Equation (10):

(10) $$\underset{j}{\mathrm{argmin}\;dist}\left(d_{j},t\right)\forall j=1..N$$

In the designed solution, *k* = 3 and the Euclidean distance metric was used. The final label of *z* was selected as the most frequent label of the *k* chosen neighbors.

#### 2.7.2. Artificial Neural Network (ANN)

The artificial neural network (ANN) is a popular class of algorithms and is based on the perceived mechanics of the human brain [43]. Standard multi-layered perceptron (MLP) was used here. The input and output layers, respectively, corresponded to the 45 features and the 5 possible classes. Hidden layers are important to model complex data, but care must be taken to avoid over-fitting. The number of the hidden layers was chosen equal to 5. The training function property ‘traingdx,’ in MATLAB [44] was used. This set the learning to gradient descent momentum and set a variable learning rate. The activation function used was the radial basis, and the maximum epochs were set to 1000.

#### 2.7.3. Support Vector Machines (SVMs)

The support vector machine (SVM) was developed by Cortes and Vapnik [45]. It searches for the optimal separating hyperplane between support vectors of two classes. The process of separating the hyperplane is described mathematically in Equation (11), where *x* is the sample vector *x* = [*x*_1_, *x*_2_…*x_p_*] with *p* attributes, *w* = [*w*_1_, *w*_2_…*w_p_*] is the weights vector, and *b* is a scalar bias.

(11) $$h\left(x\right)=sign\left(w\cdot x^{T}+b\right)$$

For categorizing multiple classes, different approaches can be used [46,47], such as the one-vs-all approach or the one-vs-one approach. In this solution, the one-vs-all strategy was used with sequential minimal optimization (SMO) to train the classifier weights. The kernel function used was the polynomial of order 3, and the regularization parameter was set to 50.

#### 2.7.4. Random Forest (RF)

The random forest (RF) was developed by using ideas put forward by Ho Tim Kam [48]. It is a technique that takes advantages of multiple classifiers. It constructs a multitude of decision trees at the training time and use the output from these trees to form a consensus. In contrast with bagging, RF may employ different decision tree techniques for the multiple subsets created. In the designed solution, the number of trees was set equal to 60, and out-of-bag predictions were retained for each tree. For the split at the nodes, the interaction-curvature method was selected, as it minimized the *p*-value of the chi-square tests of independence between each predictor and the response. The number of splits on each branch was limited to 10.

#### 2.7.5. Bagging (BG)

Bagging (BG) is a bootstrap aggregation of classification trees. Multiple trees are allowed to fit the training data so that any bias, such as over-fitting, can be dealt with by using the ensemble of trees. Hence, bagging can be a powerful tool in classification. In this study, the number of bagged trees was set to 50, and ensemble predictions were used for out-of-bag observations.

### 2.8. Performance Evaluation Metrics

#### 2.8.1. Compression Ratio

The compression ratio compares the proposed system performance in terms of reduction in the amount of information to be transmitted and classified compared to the conventional approach. In the classical case, acquired ECG data points are transmitted to the cloud without performing any features selection [24]. If $N_{r}$ and P are, respectively, the count of data points to be classified in the conventional and the proposed approach, then the compression ratio, $R_{COMP}$, can be calculated by using Equation (12):

(12) $$R_{COMP}=\frac{N_{r}}{P}$$

#### 2.8.2. Computational Complexity

The computational complexity of the embedded processing chain till the denoising module was studied in detail. The complexity of wavelet decomposition, features extraction, and cloud processing modules was analyzed at an abstract level by considering the reduction in amount of information to be processed by these modules.

The resampled signal was denoised by using an enhanced adaptive-rate FIR (ARFIR) filtering concept [27]. The arithmetic complexity of a classical *K* order FIR filter is clear from Equation (5). It executes (*K* − 1) additions and *K* multiplications while calculating an output sample. Therefore, for *N* samples, the entire computational complexity *C_FIR_* can be calculated by using Equation (13):

(13) $$C_{FIR}=\underset{Additions}{\underset{︸}{(K-1)\cdot N}}+\underset{Multiplications}{\underset{︸}{K\cdot N}}$$

For the suggested solution, the online filter selection and the selected segment resampling processes necessitated additional operations.

- Filter selection for $W^{i}$ wasresolved by using the successive approximation algorithm. Therefore, resolving the value of $Frs^{i}$ for Q reference filters, in the worst case, requires $log_{2}\left(Q\right)$ comparisons [29].
- Resampling wasrealized by using the SLI. For $W^{i}$, the complexity of SLI was $Nr^{i}$ additions and $Nr^{i}$ binary weighted right shifts. The complexity of binary weighted right shift wasnegligible compared to the addition and multiplication processes [49]. Therefore, it wasignored.
- The complexity of the $K^{i}$ order FIR filtering for $Nr^{i}$ samples could be calculated as: $\left(K^{i}-1\right)\cdot Nr^{i}$ additions and $K^{i}$·$Nr^{i}$ multiplications.

Therefore, the overall complexity of the proposed ARFIR method for $W^{i}$ can be calculated by using Equation (14):

(14) $$C_{ARFIR}=\underset{Additions}{\underset{︸}{(K^{i}-1)\cdot Nr^{i}+Nr^{i}+\log_{2}\left(Q\right)}}+\underset{Multiplications}{\underset{︸}{K^{i}\cdot Nr^{i}}}$$

#### 2.8.3. Classification Precision

The proposed solution seems appealing in terms of hardware complexity, compression, processing, and transmission efficiencies. However, it can lag in terms of precision. Therefore, the performance of the whole system wasstudied in terms of its classification accuracy. To avoid any bias in estimating the classification performance due to the limited dataset, cross-validation schema has been popularly used in the literature [47]. Therefore, 10-fold cross-validation was used in this study. All tested algorithms are provided with the same dataset, both training and test, for each fold. Similarly, to avoid any biasness in findings from any one measure, the following evaluation measures were used.

##### Accuracy (Acc)

Accuracy (Acc) is the percentage of labels that have been correctly classified. Let TP, TN, FP, and FN, respectively, denote true positives, true negatives, false positives, and false negatives in the predicted labels. Then, the mathematical formulation for accuracy is given by Equation (15). The accuracy measure results in a value between 0 and 1, with a higher value signifying a better performance.

(15) $$Accuracy=\frac{\mathrm{TP}+\mathrm{TN}}{\mathrm{TP}+\mathrm{TN}+\mathrm{FP}+\mathrm{FN}}$$

##### Normalized Mutual Information (NMI)

Normalized Mutual Information (NMI) is an information theoretic score calculated as the mutual information between two distributions-labels predicted by a classifier and the real labels of the data. The value of NMI scores ranges between 0 and 1, with a higher score signifying a better classification. It is mathematically computable by using Equation (16), where *X* is the predicted clustering labels via the algorithm, *Y* is the real labels from the data, *k*(*X*) denotes the number of clusters in *X*, ${r_{j}}^{X}$ is the number of elements in cluster *j* according to *X*, $r_{uj}$ is the number of elements predicted in *u* but actually belonging to *j*, and *n* is the total number of elements.

(16) $$MI=\frac{\sum_{1}^{k\left(X\right)}\sum_{1}^{k\left(Y\right)}r_{uj}log_{k\left(X\right)\cdot k\left(Y\right)}\left(\frac{n\cdot r_{uj}}{r_{u}^{X}r_{j}^{Y}}\right)}{\sqrt{\left(\sum_{1}^{k\left(X\right)}r_{u}^{X}log\left(\frac{r_{u}^{X}}{n}\right)\right)\left(\sum_{1}^{k\left(Y\right)}r_{u}^{Y}log\left(\frac{r_{u}^{Y}}{n}\right)\right)}}$$

##### *F*-Measure (F1)

The *F*-measure (F1) balances the values of precision and recall. We usually talk about a macro (without taking class sized into account) or micro (taking class sizes into account) *F*-measure. However, as all classes had the same data size in the studied case, we simply employ the *F*-measure. Mathematically, the *F*-measure is expressed by Equation (17), where *precision* = $\frac{TP}{\left(TP+FP\right)}$ and *recall* = $\frac{TP}{\left(TP+FN\right)}$.

(17) $$F=\frac{2*precision*recall}{precision+recall}$$

##### Kappa Index (Kappa)

The kappa index (kappa) is a widely used statistics to judge the agreement of two clustering results. It is usually considered more robust than simple accuracy because it takes the possibility of agreement by chance into account. The most popular version is the Cohen’s kappa measure, which is mathematically expressed by Equation (18), where *p*_0_ is the percentage of agreement between the predicted and actual labels, similar to accuracy, and *p_e_* is the hypothetical probabilistic chance of such an agreement occurring randomly. It is given as $p_{e}=\frac{\left(TP+TN\right)\left(TP+FN\right)+\left(FP+TN\right)\left(FP+FN\right)}{{\left(TP+TN+FP+FN\right)}^{2}}.$ A perfect classification gives *kappa* = 1, and if the classification is merely due to chance, *kappa* = 0.

(18) $$kappa=1-\frac{1-p_{0}}{1-p_{e}}$$

##### Specificity (Sp) 

Specificity (Sp) measures a test’s ability to correctly generate a negative result for instances thatdo not belong to a given class. It is also known as the “true negative” rate. It is expressed by Equation (19):

(19) $$Sp=\frac{TN}{TN+FP}$$

## 3. Results

**Figure 6:**
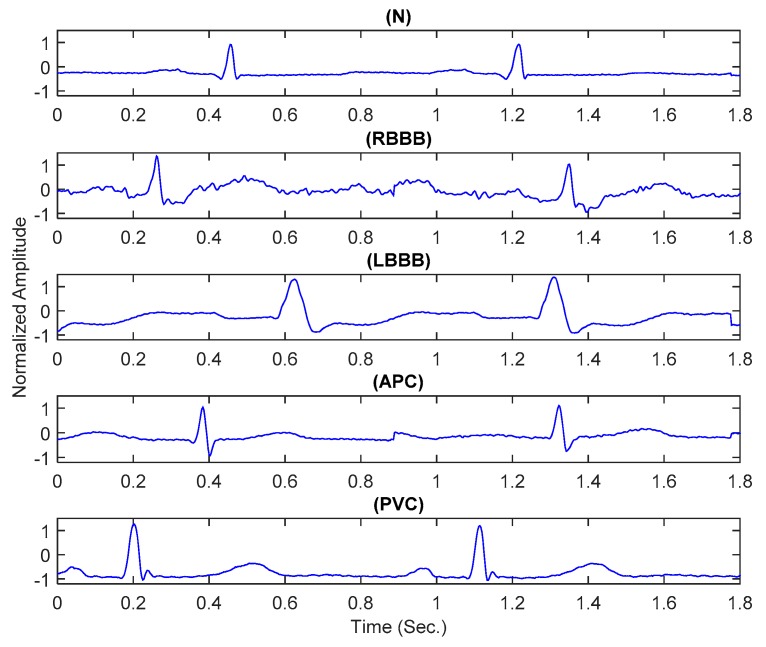
Examples of the pre-segmented electrocardiogram (ECG) signals.

The performance of the suggested solution wasstudied for five arrhythmia classes [36]. All system modules were implemented and validated by using the MATLAB^®^ [44]. Examples of the pre-segmented heartbeats from all considered classes are shown in Figure 6.

In the classical case, the signal is band-limited to 60 Hz, and it is acquired with traditional ADC of 11-bit resolution at a sampling rate of 360 Hz [38]. The signal is divided into segments of 0.9 s, and each segment consists of 320 samples.

**Figure 7:**
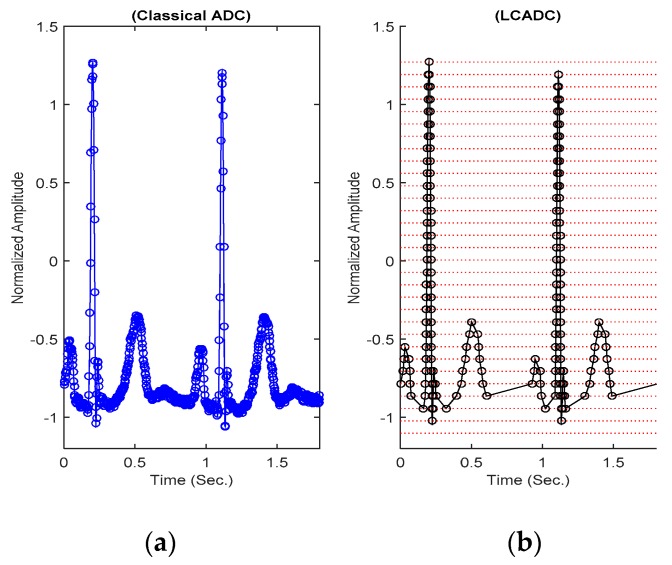
The pre-segmented ECG signal, acquired with an 11-bit resolution conventional ADC (**a**) and acquired with a 5-bit resolution LCADC (**b**).

In the proposed case, to test the LCADC, the considered ECG signals were reconstructed by using *U* = 400. The reconstructed signals were acquired with an LCADC of *M* = 5-bit. The ECG signals were band-limited up to *f_max_* = 60 Hz. Therefore, the maximum LCADC sampling frequency was $Fs_{max}$ = 3.6 kHz. Examples of the pre-segmentation ECG signal, from the PVC class, acquired with an 11-bit classical ADC and a 5-bit LCADC, are shown in Figure 7.

The LCADC output was segmented by using the ASA. The ASA adapted $L^{i}$ and $Frs^{i}$ according to the $\tilde{x}\left(t\right)$ temporal variations. It contributed to the enhancement of the system’s computational efficiency. The average compression ratios were computed for all 300 instances of each considered class. These were, respectively, 3.13-, 2.86-, 3.01-, 3.02-, and 3.05-fold for classes N, RBBB, LBBB, APC, and PVC. The attained overall average reduction in the number of collected samples for all five classes was 3.01-fold.

The resampled signal wasdenoised by using the ARFIR filtering technique [29]. A band-pass filter bank was designed for the cut-off frequencies of [*F_Cmin_* = 0.7; *F_Cmax_* = 35] Hz. The Filter bank was implemented for a set of sampling frequencies, *Fref*, between $Fs_{min}=$ 75 Hz ˃ 2·*F_Cmax_* to $F_{r}$ = 360 Hz. In this case, Δ = 19 Hz was chosen. It realized a bank of *Q* = 16 band-pass filters. A summary of the designed filter bank is shown in Table 2.

Denoising enhanced the intended signal SNR and resulted in a better classification [25]. The online filter order adaption resulted in signal denoising with a reduced computational cost compared to the time-invariant traditional ones [29]. The gains of the proposed ARFIR were computed over the classical one by using Equations (13) and (14). A summary of results for all 300 instances for each considered ECG class is presented in Table 3 and Table 4.

Table 3 and Table 4 illustrate that the proposed ARFIR technique achieved a noticeable computational gain over the traditional counterpart. It was attained by intelligently adapting the system parameters like *Frs^i^*, *Nr^i^*, and *hc_k_* for each selected segment (*W^i^*) by following the $\tilde{x}\left(t\right)$ temporal variations.

Each classifier was adjusted during the training phase with parameters set as mentioned in Section 2.7. Five classifiers, namely ANN, *k*-NN, SVM, RF, and bagging, were used. Their parameters were adjusted according to methods described in Section 2.7. They classified the intended dataset, and we measured the different evaluation metrics. The results are summarized in Table 5.

**Figure 8:**
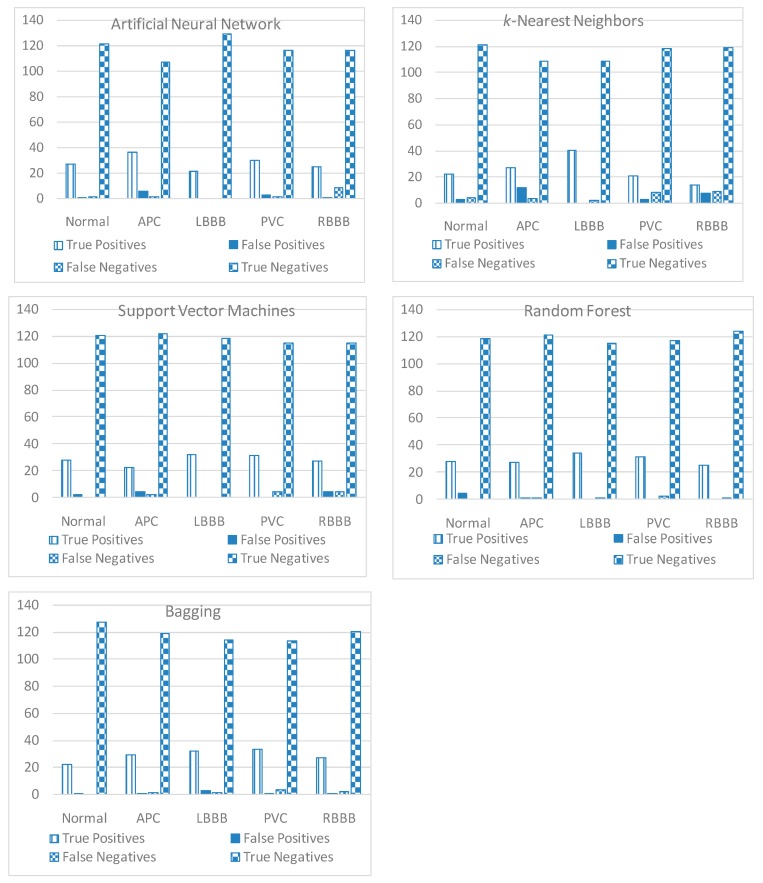
True positive (TP), false positive (FP), false negative (FN), and true negative (TN) for different ECG classes.

A summary of detail classifications results, in terms of TP, FP, FN, and TN for the five considered classes, is presented in Figure 8. These results showed that the integration of adaptive-rate signal acquisition and processing chain with RF and bagging resulted in superior classification performances as compared to *k*-NN, ANN, and SVM. Since RF uses multiple classifiers, it is less likely to be biased. This was reflected in the evaluation where RF resulted in the best NMI score and kappa statistics. On the other hand, *k*-NN can be easily biased by the chosen neighbors, particularly if there are outliers in the data. This was reflected in its low score across all indices. Overall, the highest accuracy score of 0.97 was attained by the designed solution.

For further analysis, we considered the detail classification results for each intended class, as shown in Figure 8. The overall false positive and false negative counts by using RF were the lowest compared to bagging, k-NN, ANN, and SVM, thus confirming the outperformance of RF compared to other considered algorithms. The most difficult class to discriminate was the RBBB, which was confused with LBBB, while the easiest class to discriminate was normal compared to the other four classes.

## 4. Discussion

The appealing features of the suggested solution are clear from results, presented in Section 3. This solution was attained by intelligently exploiting level-crossing sampling, adaptive-rate processing, and robust classifiers. The values of $Nr^{i}$ were adapted by the ASA. We showed how $Fs^{i}$, $L^{i}$, and $Frs^{i}$ were adjusted as a function of the temporal variations of $\tilde{x}\left(t\right)$. The adjustment of $Frs^{i}$ avoided unnecessary interpolations during the resampling process [29]. This signal-driven, adaptive-rate sampling resulted in a three-fold decrease in the number of collected samples compared to classical counterparts. It guaranteed a noticeable reduction in the processing load of the post denoising and features extraction.

$K^{i}$ represents how the adjustment of *hc_k_* for *W^i^* avoids the unnecessary operations while conditioning the selected segments. It yielded a noteworthy computational gain of the proposed denoising method over the conventional counterparts. The average gains in additions and multiplications, for the considered 1500 instants, of the employed ARFIR over the conventional one were 7.81- and 7.99-fold, respectively. Additionally, the adaptation of $Frs^{i}$ resulted in an adaptive-rate sub-band decomposition. $Frs^{i}$ reflected the $\tilde{x}\left(t\right)$ frequency content [35]. Therefore, it was able to result in an improved focus on the band of interest, as the decomposition process is a function of $Frs^{i}$ [40]. The decomposition was attained by using the half-band FIR filters. A three-fold decrease in the number of collected samples confirmed computationally efficient sub-band decomposition compared to the counter fix-rate solutions [4,6,12,13,29,40].

Originally, each intended instance was composed of 320 samples. After features extraction, their dimensionality was reduced to 45 features, resulting in a 7.1-fold dimensionality reduction and guaranteeing the same factor of reduction in the data transmission activity, bandwidth, and power consumption. Additionally, on the cloud side, the processing of 7.1-fold less amount of information assured a similar factor of processing and resource utilization gain during the classification process.

The adoption of performing most of the signal processing tasks via the front-end processor and transmitting only the extracted features to the cloud was also beneficial in realizing an optimized front-end ECG wearable device while keeping the system configurable. Moreover, in the designed framework, the signal was digitized with a M= 5-bit resolution LCADC. In the classical case, the digitization is performed with an M= 11-bit resolution ADC [4,5,6,12,13,14,15]. Our study confirmed a simpler circuit level realization compared to the traditions ones. A low resolution ADC can also be used in the classical case, but it can reduce the system SNR [38] and can therefore degrade classification performance. On other hand, the effective resolution of LCADC is fairly independent of M [38]. Therefore, an accuracy of 97% was achieved by the proposed solution, even when using a 5-bit resolution LCADC.

The idea of embedding the level-crossing sampling and adaptive-rate processing in ECG signal-based automatic arrhythmia diagnosis is a novel concept. In [16,24,30,31,32], new approaches were proposed for effective ECG acquisition. These mainly focused on the analog-to-digital conversion of ECG signals. In [30,31], the authors demonstrated that how the use of LCS-based architectures led towards a simplified and power efficient analog/digital (A/D) conversion. In [32], the authors showed how the proposed ADC could adjust its effective resolution as a function of the activity of the input signal. In [16,24], efficient and real-time QRS detection mechanisms were proposed.

In contrast to [16,24,30,31,32], the proposed solution did not only focus on the design of an effective LCADC for ECG signal acquisition or QRS complexes detection; it additionally presented an application of the ASA and the adaptive-rate resampling, denoising, and sub-band decomposition approaches on the LCADC output. It was performed to achieve efficient segmentation, noise reduction, and features extraction of digitized ECG pulses to well prepare them for post cloud-based automatic arrhythmia classification.

The proposed technique is original, and comparing it with existing state-of-the-art methods was not straight forward, since they are based on classical sampling and processing. Additionally, each study uses a different number of subjects and different types of arrhythmia classes. It is also delicate to compare the diversity of classification methods and techniques for ECG signal processing. However, a comparison was performed with previous studies that use the same ECG dataset and DWT-based features extraction. The highest accuracies of classification for all considered studies are presented in Table 6, which shows that the proposed solution attained an analogous or better classification accuracy as compared to the fix-rate counterparts [4,5,6,12,13,14,15] while assuring noticeable processing, transmission, and hardware simplicity gains.

The main advantage of the proposed solution over the previous ones is the elimination of unnecessary samples to process and to introduce a real-time compression gain in the system. This was achieved by tactfully embedding the level-crossing sampling and ASA into the system. Similar gains could be achieved by embedding these concepts in counterparts [4,5,6,12,13,14,15].

## 5. Conclusions

An original level-crossing ECG signal sampling, adaptive-rate denoising, and sub-band decomposition and features extraction approach was proposed. It was shown that the proposed framework achieved a three-fold reduction in the number of acquired samples, thus leading to a significant reduction in the computational complexity of the designed system compared to the conventional counterparts. The overall average gains in additions and multiplications for the designed adaptive-rate denoising module were computed as 7.81- and 7.99-fold, respectively. The adaptive-rate processing promised a similar factor of processing gain during the adaptive-rate sub-band decomposition. The proposed features extraction concept reduced the incoming data dimensionality with a factor of 7.1 and assured a remarkable gain in terms of the power consumption and transmission activity between the ECG wearable device and cloud-based classification. Moreover, a similar magnitude of processing gain was also evident in the post cloud-based classifier because it had to deal with a 7.1 times lesser amount of information. The highest classification accuracy of 97% was attained by the random forest, which is comparable, and in some cases better, to existing state-of-the-art solutions. The plus side is our gain in compression, processing, and transmission bandwidth, which show the potential of using the suggested solution for the design and development of low power and efficient ECG wearable devices in the mobile healthcare framework.

## Figures and Tables

**Table 1:** MIT-BIH arrhythmia records used in this study. RBBB: right bundle branch block; LBBB: left bundle branch block; APC: atrial premature contraction; PVC: premature ventricular contraction.

CLASS	Records	Number of Beats Used
**Normal**	116, 119, 209	300
**RBBB**	118, 124, 212	300
**LBBB**	109, 111, 214	300
**APC**	118, 200, 209	300
**PVC**	119, 200, 233	300

**Table 2:** Summary of the reference filters bank parameters.

hc_k_	h1_k_	h2_k_	h3_k_	h4_k_	h5_k_	h6_k_	h7_k_	h8_k_	h9_k_	h10_k_	h11_k_	h12_k_	h13_k_	h14_k_	h15_k_	h16_k_
***Fref_c_* (Hz)**	75	94	113	132	151	170	189	208	227	246	265	284	303	322	341	360
***K_c_***	23	30	36	43	49	55	61	68	74	80	86	92	99	105	111	117

**Table 3:** Summary of the adaptive-rate finite impulse response (ARFIR) computational gains, in additions, over the classical FIR filtering.

CLASS	Max Gain in Additions	Min Gain in Additions	Mean Gain in Additions	Median Gain in Additions
**Normal**	15.97	6.67	8.12	7.90
**RBBB**	12.17	6.66	7.32	7.27
**LBBB**	12.46	6.70	7.86	7.66
**APC**	10.75	6.75	7.71	7.56
**PVC**	15.06	6.71	8.03	7.77

**Table 4:** Summary of the ARFIR computational gains, in multiplications, over the classical FIR filtering.

CLASS	Max Gain in Multiplications	Min Gain in Multiplications	Mean Gain in Multiplications	Median Gain in Multiplications
**Normal**	16.14	6.74	8.31	8.09
**RBBB**	12.30	6.73	7.48	7.43
**LBBB**	12.58	6.76	8.04	7.84
**APC**	10.86	6.81	7.89	7.74
**PVC**	15.22	6.76	8.22	7.95

**Table 5:** Performance of different classifiers using five evaluation metrics on the test dataset. ANN: artificial neural network; *k*-NN: *k*-nearest neighbors; SVM: support vector machine; and RF: random forest.

Classifier	Acc	NMI	F1	Kappa	Sp
**ANN**	0.93	0.82	0.93	0.77	0.98
***k*-NN**	0.83	0.71	0.81	0.46	0.96
**SVM**	0.93	0.84	0.93	0.75	0.98
**RF**	0.97	0.94	0.97	0.90	0.99
**Bagging**	0.96	0.89	0.96	0.79	0.99

**Table 6:** Comparison with state-of-the-art methods.

Study	Features Extraction	Classification Method	No. of Classes	Accuracy (%)
[4]	Wavelet Packet Decomposition (WPD) and Wavelet-Based kernel Principle Component Analysis (wkPCA)	Backpropagation Neural Network (BNN)	**5**	**98.03**
[5]	Wavelet Packet Entropy (WPE)	Random Forests (RF)	**5**	**94.61**
[6]	Discrete Wavelet Transform (DWT)	Probabilistic Neural Network (PNN)	**8**	**92.75**
[12]	Discrete Wavelet Transform (DWT), Temporal, and Morphological	Support Vector Machine (SVM)	**4**	**98.39**
[13]	Discrete Wavelet Transform (DWT) and Principle Component Analysis (PCA)	Support Vector Machine (SVM)-Radial Basis Function (RBF)	**5**	**96.92**
[14]	Bispectrumand Principle Component Analysis (PCA)	Support Vector Machine (SVM)-Radial Basis Function (RBF)	**5**	**93.48**
[15]	HermiteFunction Coefficient and Temporal Features	Optimized block-based Neural Network (OBNN)	**5**	**97.00**
This Study	Wavelet Decomposition and Sub-band statistical features	Random Forests (RF)	5	97.00
